# Patterns and Determinants of Sexual Assault Cases Undergoing Medical Examination: Evidence From a Tertiary Care Center in Rajasthan, India

**DOI:** 10.7759/cureus.108730

**Published:** 2026-05-12

**Authors:** Chetan Kumar R., Kalpeshkumar C Patani, Jaskirat K Hundal, Sajidali S Saiyad, Prasanta Chatterjee Biswas, Gnanadesigan Ekambaram, Alkeshkumar R Vara, Sandip Sendhav

**Affiliations:** 1 Department of Forensic Medicine, Rajmata Vijaya Raje Scindia Medical College, Bhilwara, Bhilwara, IND; 2 Department of Forensic Medicine and Toxicology, Nootan Medical College and Research Centre, Visnagar, Sankalchand Patel University, Visnagar, IND; 3 Department of Clinical Sciences, Washington University of Health and Sciences, San Pedro, BLZ; 4 Department of Physiology, Pacific Medical College and Hospital, Pacific Medical University, Udaipur, IND; 5 Department of Biostatistics, Parul University, Vadodara, IND; 6 Department of Physiology, Nootan Medical College and Research Centre, Visnagar, Sankalchand Patel University, Visnagar, IND; 7 Department of Biochemistry, Nootan Medical College and Research Centre, Visnagar, Sankalchand Patel University, Visnagar, IND

**Keywords:** adolescent victims, delayed reporting, forensic evidence, gender-based violence, injury patterns, intimate partner violence, medico-legal examination, rural-urban disparities, sexual assault, victim-perpetrator relationship

## Abstract

Background: Sexual assault is a major public health and medico-legal concern, particularly in low- and middle-income settings where underreporting and sociocultural barriers limit timely access to care and justice.

Objective: To analyze the socio-demographic, clinical, and contextual determinants of sexual assault cases undergoing medico-legal examination at a tertiary care center in Rajasthan.

Methods: A retrospective observational study was conducted using medico-legal records of 905 cases examined between 2023 and 2025. Data on victim characteristics, assault circumstances, clinical findings, and legal variables were extracted and analyzed. Descriptive statistics were used to summarize findings, and multivariate logistic regression was applied to identify predictors of delayed reporting (>72 hours).

Results: Adolescents aged 12-18 years constituted the majority of victims (468/905, 51.7%), with a predominance of unmarried individuals (677/905, 74.8%) and those from lower socioeconomic strata. Delayed reporting was observed in 516/905 (57.0%) cases. Most assaults were perpetrated by known individuals (779/905, 86.1%) and occurred predominantly during nighttime (579/905, 64.0%). Physical injuries were absent in 842/905 (93.0%) cases, while semen detection was reported in 217/905 (24.0%). Multivariate analysis identified rural residence (adjusted odds ratio (AOR): 1.9; 95% confidence interval (CI): 1.3-2.7; p = 0.002), known assailant (AOR: 2.5; 95% CI: 1.7-3.8; p < 0.001), family pressure (AOR: 3.2; 95% CI: 2.1-4.9; p < 0.001), and minor age (AOR: 1.6; 95% CI: 1.1-2.3; p = 0.01) as significant predictors of delayed reporting.

Conclusion: The findings demonstrate a complex interplay of demographic vulnerability, interpersonal relationships, and sociocultural factors influencing sexual assault dynamics. The study provides a multidimensional perspective by integrating clinical, behavioral, and legal variables with analytical modeling. Strengthening early reporting, improving access to trauma-informed care, and addressing sociocultural barriers are essential to enhance medico-legal outcomes and survivor support.

## Introduction

Sexual assault is a major medico-legal and public health concern with significant physical, psychological, and social consequences for survivors. It encompasses a range of non-consensual sexual acts and disproportionately affects women and children, although individuals of all genders may be impacted. Beyond immediate physical trauma, survivors frequently experience long-term mental health consequences, including depression and post-traumatic stress disorder, highlighting the need for comprehensive and timely interventions [1,2]. In low- and middle-income countries such as India, the burden is further compounded by underreporting, sociocultural stigma, and limited access to survivor-centered services.

Medico-legal examination plays a critical role in the management of sexual assault cases by facilitating clinical care and supporting legal proceedings through documentation of injuries and collection of forensic evidence. Timely examination is essential; however, delays in reporting are common and often result in loss of crucial evidence and reduced effectiveness of medical and legal interventions [3]. Studies have shown that trauma-informed and empathetic care can significantly improve survivor engagement with healthcare and judicial systems, thereby enhancing recovery and access to justice [4].

Socio-demographic characteristics of victims provide important insights into vulnerability patterns. Previous studies from different regions of India have consistently reported that adolescents and young adults constitute the majority of victims, with a predominance of unmarried individuals and those from lower socioeconomic backgrounds [5,6]. Additionally, most assaults are committed by individuals known to the victim, including intimate partners, family members, and acquaintances, reflecting complex interpersonal dynamics [7]. These patterns emphasize the importance of contextualizing sexual assault within broader social and behavioral frameworks.

Hospital-based studies from Rajasthan and other parts of India have identified recurring trends such as delayed reporting beyond 72 hours, predominance of young victims, and low prevalence of detectable injuries at the time of examination [5,8]. While these studies provide valuable descriptive data, they are limited in their ability to explain underlying determinants of reporting behavior and clinical findings.

Recent research highlights the role of behavioral and contextual factors, such as fear of the accused, familial pressure, and social stigma, in influencing reporting patterns [9]. Similarly, characteristics of the accused, including relationship with the victim and substance use, have been associated with variations in assault dynamics and reporting delays [10]. However, integration of these variables into comprehensive analytical models remains limited in existing literature.

Therefore, the present study aimed to evaluate the socio-demographic, medico-legal, and contextual characteristics of sexual assault survivors undergoing medico-legal examination at a tertiary care center in Rajasthan, with particular emphasis on factors associated with delayed reporting - an aspect underexplored in prior regional studies. It examines socio-demographic characteristics, reporting intervals, and clinical and forensic findings, and further identifies predictors of delayed reporting using multivariate analysis. By integrating these dimensions, the study seeks to provide a comprehensive understanding of sexual assault dynamics in the regional context. The findings are expected to inform clinical practice, strengthen medico-legal processes, and guide policy interventions aimed at improving survivor care.

## Materials and methods

Study design and setting

This retrospective, hospital-based observational study was conducted in the Department of Forensic Medicine and Toxicology at Mahatma Gandhi Hospital, Bhilwara, Rajasthan, India. The study was designed to analyze the socio-demographic, clinical, and contextual determinants of sexual assault cases undergoing medico-legal examination in a tertiary care setting. A retrospective approach was adopted to enable systematic evaluation of routinely maintained medico-legal records over a defined time period, allowing identification of patterns and associated factors in real-world clinical conditions.

Study population

The study included all individuals presenting for medico-legal examination following alleged sexual assault during the study period, irrespective of age, at the institution over a three-year period from January 2023 to December 2025, with retrospective data extraction performed in early 2026. A total of 905 cases were included in the final analysis. Cases with incomplete or missing essential information were excluded from specific analyses where necessary, but were retained for overall descriptive evaluation to maintain the integrity of the dataset.

Data collection and processing

Data were extracted retrospectively from medico-legal examination records maintained in the Department of Forensic Medicine using a structured data abstraction format. Information collected included socio-demographic variables, assault characteristics, reporting patterns, medico-legal findings, and selected healthcare-system variables.

Socio-demographic variables included age, place of residence (urban/rural), marital status, occupation, and socioeconomic category. Socioeconomic status was categorized using available socioeconomic indicators documented in medico-legal records, including occupation-related and income-related information where available. Since educational status was inconsistently documented in retrospective records, a formally validated composite socioeconomic scale was not applied.

Assault-related variables included the relationship between survivor and accused, place of occurrence, timing of assault, involvement of multiple perpetrators, and alleged alcohol use by the accused. Relationship with the accused was categorized as intimate partner/boyfriend, family member, neighbor, friend/acquaintance, or stranger. The place of occurrence was categorized as home, isolated area, workplace, or other locations, while the timing of the incident was classified as day or night.

The time interval between the alleged assault and medico-legal examination was documented and categorized as less than 24 hours, 24-72 hours, or more than 72 hours. Delayed reporting was operationally defined as presentation for medico-legal examination more than 72 hours after the alleged incident and was considered the primary analytical outcome variable for regression analysis.

Reasons for delayed reporting were extracted from medico-legal records based on survivor history, police requisition details, and clinician documentation available at the time of examination. Where documented, reasons for delay were categorized into fear of the accused, family pressure, social stigma, lack of transport, and police-related factors. Only the primary documented reason for delay was considered for analysis.

Clinical and forensic variables included the presence or absence of genital injuries, non-genital injuries, signs of struggle, semen detection status, and the nature of injuries documented during examination. Injury findings were categorized as genital injury, body injury, combined genital and body injury, or absence of detectable injury. Legal and healthcare-system variables included timing of First Information Report (FIR) registration, reporting source, and consent for medico-legal examination.

Outcome measures

The primary outcome measure was delayed reporting, defined as presentation for medico-legal examination more than 72 hours after the incident. Secondary outcomes included the presence of injuries and the distribution of socio-demographic characteristics.

Statistical analysis

Statistical analysis was performed using IBM SPSS Statistics for Windows, Version 26 (Released 2018; IBM Corp., Armonk, New York, United States). Descriptive statistics were used to summarize categorical variables in terms of frequencies and percentages. Data are presented as frequency (n (%)), unless otherwise specified.

Associations between selected independent variables and delayed reporting were initially assessed using a chi-square test or Fisher’s exact test, as appropriate. Univariate logistic regression analysis was subsequently performed to estimate crude odds ratios (ORs) for variables associated with delayed reporting. Variables included in the multivariate logistic regression model comprised age group, place of residence, relationship with the accused, and family-related reporting barriers, as these variables were considered clinically relevant, contextually important based on previous literature, and sufficiently documented in the study records. Variables with substantial missing or inconsistent documentation were not included in the final regression model.

Multivariate logistic regression analysis was performed to estimate adjusted odds ratios (AORs) with 95% confidence intervals (CIs) for factors associated with delayed reporting. Model fit was assessed using the Hosmer-Lemeshow goodness-of-fit test and Nagelkerke R^2^. Multicollinearity among variables was evaluated using the variance inflation factor (VIF). Statistical significance was considered at p < 0.05.

Ethical considerations

The study was conducted after obtaining approval from the Rajmata Vijaya Raje Scindia Government Medical College Ethics and Research Committee with approval number RVRSMC/IEC/2026/16. All data were anonymized to ensure the confidentiality and privacy of the victims. As the study was based on retrospective record review, the requirement for informed consent was waived in accordance with institutional guidelines.

## Results

A total of 905 alleged cases of sexual assault undergoing medico-legal examination were included in the study.

Socio-demographic characteristics

The socio-demographic profile of victims is summarized in Table 1. The majority of victims belonged to the 12-18 years age group (468, 51.7%), followed by 19-30 years (278, 30.7%). Victims aged less than 12 years constituted 13 (1.4%), while those above 40 years accounted for 25 (2.8%). Urban residents comprised 490 (54.1%) of cases, whereas 415 (45.9%) were from rural areas. A substantial proportion of victims were unmarried (677, 74.8%), followed by married individuals (212, 23.4%).

**Table 1:** Socio-Demographic Profile of Victims (n = 905) Values are presented as frequency (n) and percentage (%). Descriptive statistics were used for categorical variables.

Variable	Category	n (%)
Age group (years)	<12	13 (1.4)
	12-18	468 (51.7)
	19-30	278 (30.7)
	31-40	121 (13.4)
	>40	25 (2.8)
Residence	Urban	490 (54.1)
	Rural	415 (45.9)
Marital status	Unmarried	677 (74.8)
	Married	212 (23.4)
	Divorced	9 (1.0)
	Widow	7 (0.8)
Occupation	Student	416 (46.0)
	Unemployed/housewife	278 (30.7)
	Laborer	115 (12.7)
	Service/professional	60 (6.6)
	Others	36 (4.0)
Socioeconomic status	Upper	54 (6.0)
	Upper middle	127 (14.0)
	Lower middle	235 (26.0)
	Upper lower	338 (37.3)
	Lower	151 (16.7)

With respect to occupation, students formed the largest group (416, 46.0%), followed by unemployed individuals or housewives (278, 30.7%). Socioeconomic status assessment revealed that the majority of victims belonged to the upper lower socioeconomic class (338, 37.3%) and lower middle socioeconomic class (235, 26.0%).

Assault characteristics and reporting pattern

The characteristics of the assault and reporting patterns are summarized in Table 2. A majority of victims (516, 57.0%) presented for medico-legal examination more than 72 hours after the incident, while 235 (26.0%) reported within 24-72 hours and 154 (17.0%) within 24 hours.

**Table 2:** Assault Characteristics and Reporting Pattern (n = 905) Values are expressed as frequency (n) and percentage (%). * Reason for delay analysis was restricted to delayed reporting cases (>72 hours) (n = 516). Only the primary documented reason for delay was considered for analysis. Delayed reporting was defined as presentation for medico-legal examination more than 72 hours after the incident.

Variable	Category	n (%)
Time to examination	<24 hours	154 (17.0)
	24-72 hours	235 (26.0)
	>72 hours	516 (57.0)
Reason for delay*	Fear of the accused	187 (36.2)
	Family pressure	145 (28.1)
	Social stigma	109 (21.1)
	Lack of transport	60 (11.6)
	Police hesitation	15 (2.9)
Place of assault	Home	290 (32.0)
	Isolated area	398 (44.0)
	Workplace	127 (14.0)
	Others	90 (10.0)
Time of incident	Day	326 (36.0)
	Night	579 (64.0)

Among cases with delayed reporting (>72 hours) (n = 516), fear of the accused was the most commonly documented reason (187, 36.2%), followed by family pressure (145, 28.1%) and social stigma (109, 21.1%). Most incidents occurred in isolated areas (398, 44.0%) or at home (290, 32.0%), and a majority took place during nighttime (579, 64.0%).

Relationship and accused characteristics

Details regarding the relationship between victims and accused, along with characteristics of the accused, are presented in Table 3. The most common category was intimate partner (290, 32.0%), followed by acquaintances (199, 22.0%) and neighbors (163, 18.0%). Family members (127, 14.0%) and strangers (126, 14.0%) accounted for a comparable proportion of cases.

**Table 3:** Relationship and Accused Characteristics (n = 905) Values are presented as frequency (n) and percentage (%). Alcohol use refers to alleged alcohol consumption by the accused at the time of assault.

Variable	Category	n (%)
Relationship with the accused	Boyfriend/intimate partner	290 (32.0)
	Family member	127 (14.0)
	Neighbor	163 (18.0)
	Friend/acquaintance	199 (22.0)
	Stranger	126 (14.0)
Age of the accused	<25 years	332 (36.7)
	25-40 years	423 (46.7)
	>40 years	150 (16.6)
Alcohol use by the accused	Yes	296 (32.7)
	No	609 (67.3)
Multiple perpetrators	Yes	109 (12.0)
	No	796 (88.0)

The majority of accused individuals were aged 25-40 years (423, 46.7%). Alcohol use by the accused was reported in 296 (32.7%) cases, and multiple perpetrators were involved in 109 (12.0%) cases.

Clinical and forensic findings

Clinical and forensic findings are summarized in Table 4. The majority of victims (842, 93.0%) had no detectable physical injuries at the time of examination. Comparatively higher frequencies of documented injuries and signs of struggle were observed among unmarried survivors than among married survivors. Genital injuries were observed in 28 (3.1%) cases, while non-genital injuries were present in 20 (2.2%) cases.

**Table 4:** Clinical and Forensic Findings (n = 905) Values are expressed as frequency (n) and percentage (%). The injury pattern was categorized according to medico-legal examination findings. Semen detection was based on available forensic laboratory reports.

Variable	Category	n (%)
Injury pattern	No injury	842 (93.0)
	Genital injury	28 (3.1)
	Body injury	20 (2.2)
	Both genital and body injury	15 (1.7)
Semen detection	Present	217 (24.0)
	Absent	688 (76.0)
Signs of struggle	Present	253 (28.0)
	Absent	652 (72.0)

Semen detection was reported in 217 (24.0%) cases and was comparatively more common among survivors presenting within 72 hours of assault than among delayed reporting cases (>72 hours), and signs of struggle were present in 253 (28.0%) cases.

Legal and system variables

Legal and system-related variables are presented in Table 5. In most cases, the FIR was registered before the medico-legal examination (634, 70.1%). The police were the primary source of reporting (543, 60.0%), followed by family members (235, 26.0%). Consent for examination was obtained in 833 (92.0%) cases.

**Table 5:** Legal and System Variables (n = 905) Values are presented as frequency (n) and percentage (%). FIR: First Information Report; NGO: non-governmental organization

Variable	Category	n (%)
FIR timing	Before examination	634 (70.1)
	After examination	271 (29.9)
Reporting source	Police	543 (60.0)
	Family	235 (26.0)
	Self/NGO	127 (14.0)
Consent for examination	Given	833 (92.0)
	Refused	72 (8.0)

Bivariate analysis

Bivariate analysis demonstrated significant associations between delayed reporting and rural residence, assaults involving known accused individuals, minor age, and family-related reporting barriers (Table 6). Variables demonstrating clinical relevance and statistical association in univariate analysis were subsequently included in the multivariate logistic regression model.

**Table 6:** Bivariate Analysis of Factors Associated With Delayed Reporting (>72 Hours) Data are presented as frequency, n (%). Associations between categorical variables and delayed reporting (>72 hours) were assessed using Pearson’s chi-square test. Crude odds ratios (ORs) with 95% confidence intervals (CIs) were estimated using univariate logistic regression analysis. Statistical significance was considered at p < 0.05. * Known accused includes intimate partner, family member, neighbor, and acquaintance categories.

Variable	Delayed Reporting Present, n (%)	Delayed Reporting Absent, n (%)	Crude OR (95% CI)	χ^2^ value	p-value
Rural residence	278 (66.9)	137 (33.1)	1.82 (1.38-2.40)	10.84	0.001
Known accused individuals*	472 (60.6)	307 (39.4)	2.31 (1.63-3.28)	18.27	<0.001
Minor age (<18 years)	312 (64.9)	169 (35.1)	1.58 (1.18-2.12)	7.12	0.008
Family-related reporting barriers	145 (28.1)	0 (0)	2.94 (1.97-4.37)	24.63	<0.001

Multivariate analysis

Multivariate logistic regression analysis identified several factors significantly associated with delayed reporting (Table 7). Rural residence was associated with higher odds of delayed reporting (AOR = 1.9, 95% CI: 1.3-2.7, Wald χ^2^ = 9.41, p = 0.002). Assaults involving known accused individuals were also associated with increased odds of delayed reporting (AOR = 2.5, 95% CI: 1.7-3.8, Wald χ^2^ = 18.62, p < 0.001).

**Table 7:** Multivariate Logistic Regression Analysis for Predictors of Delayed Reporting (>72 Hours) Multivariate logistic regression analysis was performed to identify predictors of delayed reporting (>72 hours). Adjusted odds ratios (AORs) with 95% confidence intervals (CIs), Wald chi-square (χ^2^) statistics, degrees of freedom (df), and p-values are presented. Wald χ^2^ statistics were used to assess the significance of individual predictors in the regression model. The model demonstrated acceptable goodness-of-fit (Hosmer-Lemeshow χ^2^ = 8.62, df = 8, p = 0.38) with moderate explanatory power (Nagelkerke R^2^ = 0.214). Statistical significance was considered at p < 0.05.

Predictor	AOR	95% CI	Wald Chi-Square (χ^2^) (df = 1)	p-value
Rural residence	1.9	1.3-2.7	9.41	0.002
Known accused individuals	2.5	1.7-3.8	18.62	<0.001
Family pressure	3.2	2.1-4.9	24.15	<0.001
Minor age	1.6	1.1-2.3	6.53	0.01

Family-related reporting barriers demonstrated a significant statistical association with delayed reporting (AOR = 3.2, 95% CI: 2.1-4.9, Wald χ^2^ = 24.15, p < 0.001). Additionally, victims below 18 years had higher odds of delayed reporting compared to adults (AOR = 1.6, 95% CI: 1.1-2.3, Wald χ^2^ = 6.53, p = 0.01). The logistic regression model demonstrated acceptable goodness-of-fit (Hosmer-Lemeshow χ^2^ = 8.62, df = 8, p = 0.38) with moderate explanatory power (Nagelkerke R^2^ = 0.214).

## Discussion

The present study provides a comprehensive analysis of socio-demographic, clinical, and contextual determinants of sexual assault cases undergoing medico-legal examination in a tertiary care setting in Rajasthan. The findings reveal consistent patterns in victim characteristics, reporting behavior, and medico-legal outcomes, while also highlighting important contextual determinants that influence reporting and evidence documentation.

The predominance of adolescent victims observed in this study is consistent with both national and global trends, indicating heightened vulnerability among younger individuals [1,4,5]. Adolescents are often exposed to unequal power dynamics, limited autonomy, and evolving social relationships, which may increase susceptibility to coercion and abuse. Similar findings have been reported in Indian hospital-based studies, where adolescents constitute the largest proportion of victims [4,5]. Global estimates further corroborate that younger women are disproportionately affected by sexual violence [1]. This emphasizes the need for targeted preventive strategies focusing on adolescents and young adults.

The observed urban predominance contrasts with earlier regional studies that reported higher rural representation [8]. This difference may partly reflect referral and healthcare-accessibility patterns inherent to tertiary-care hospital-based studies. Survivors residing in urban settings are more likely to access medico-legal services because of closer proximity to healthcare institutions, improved transportation, greater legal awareness, and easier interaction with police and judicial systems. In contrast, rural survivors may remain underrepresented because of structural barriers, including limited healthcare access, transportation delays, sociocultural stigma, and reduced reporting opportunities. Consequently, the urban predominance observed in the present study should not necessarily be interpreted as a higher true incidence in urban populations, but rather as a reflection of differential healthcare utilization and reporting behavior. Urban populations may have better access to medico-legal services and greater awareness of legal rights, leading to increased reporting. Conversely, underreporting in rural areas due to stigma, fear of social repercussions, and limited institutional access remains a critical concern, as also noted in previous literature [11,12]. The concentration of cases in lower socioeconomic strata further supports the role of structural inequalities as determinants of vulnerability [5,6].

Delayed reporting emerged as a major finding, with more than half of the victims presenting after 72 hours. This is consistent with earlier Indian studies and global observations [5,8,13]. The high proportion of delayed reporting observed in the present study may have influenced the medico-legal detection of physical injuries and biological evidence, as delayed presentation is known to reduce forensic evidence recovery in sexual assault evaluations [3]. Although detailed subgroup analysis according to reporting interval was limited in the current dataset, the findings remain consistent with established forensic literature. The reasons demonstrated in this study, particularly fear of the accused, family pressure, and social stigma, align with established evidence highlighting sociocultural barriers to disclosure [9,13]. These findings reinforce the need for community-level interventions aimed at reducing stigma and promoting early reporting.

The majority of assaults were perpetrated by individuals known to the victim, including intimate partners, acquaintances, and family members. This finding is consistent with extensive literature indicating that sexual violence is predominantly an interpersonal phenomenon rather than a stranger-driven crime [7,14]. The high proportion of intimate partner involvement underscores the intersection between sexual violence and relationship dynamics, including issues of consent, coercion, and gender norms. These findings challenge prevailing societal misconceptions and highlight the importance of preventive strategies that address violence within known social networks.

Clinical findings in the present study indicate that a large proportion of victims had no detectable physical injuries at the time of examination. This observation is consistent with prior studies, which emphasize that the absence of injuries does not negate the occurrence of sexual assault [3]. Factors such as delayed reporting, lack of overt resistance, and non-violent coercion contribute to this pattern. Similarly, the relatively low rate of semen detection is likely attributable to delayed examination and biological degradation of evidence. These findings underscore the limitations of relying solely on physical evidence and highlight the importance of comprehensive medico-legal evaluation.

The comparatively higher prevalence of documented injuries and signs of struggle among unmarried survivors may reflect differences in assault dynamics, resistance patterns, sociocultural circumstances, and timing of medico-legal presentation. However, these observations should be interpreted cautiously because detailed subgroup analysis according to marital status and reporting interval was limited by the retrospective nature of the dataset.

The relatively low prevalence of documented physical injuries observed in the present study may reflect delayed medico-legal presentation, variability in assault dynamics, healing of minor injuries prior to examination, and the recognized possibility of absent visible injuries despite confirmed sexual assault. Similar observations have been reported in forensic literature, emphasizing that the absence of injuries does not exclude sexual assault.

The association between alcohol use by the accused and sexual assault observed in this study is supported by existing literature demonstrating a strong link between substance use and aggressive or coercive behavior [10,11]. Alcohol consumption may impair judgment and reduce inhibitions, thereby increasing the likelihood of perpetration. The presence of multiple perpetrators in a subset of cases further indicates the severity and complexity of certain assault scenarios, necessitating enhanced forensic and legal scrutiny.

The analysis of legal and system variables indicates that most cases were reported through police channels, with FIR registration preceding medical examination in the majority of instances. This reflects adherence to established medico-legal protocols. However, the presence of cases reported through family or self-referral highlights variability in reporting pathways and potential gaps in access to formal systems. High rates of consent for examination suggest compliance with ethical standards. Although refusal was documented in 8.0% of cases, this finding highlights the importance of trauma-informed approaches to mitigate survivor distress and ensure informed decision-making during medico-legal procedures.

Multivariate analysis demonstrated rural residence, involvement of known assailants, family pressure, and younger age as significant predictors of reporting delay. These findings are consistent with existing evidence that emphasizes the role of social proximity and sociocultural constraints in influencing disclosure patterns [9,13]. The identification of these predictors has important implications for targeted interventions, including strengthening community awareness, improving accessibility of medico-legal services, and enhancing support mechanisms for vulnerable groups.

From a theoretical perspective, the findings align with the socio-ecological model of violence, which highlights the interplay of individual, relational, community, and societal factors [15,16]. The observed associations between demographic vulnerability, interpersonal relationships, and reporting behavior reflect the multifactorial nature of sexual assault. Practically, these results underscore the need for integrated, multi-sectoral approaches involving healthcare, legal systems, and community-based interventions.

The strengths of this study include a relatively large sample size and the integration of socio-demographic, clinical, behavioral, and legal variables. Additionally, the use of multivariate analysis to identify independent predictors provides additional analytical insight into factors associated with delayed reporting.

The present study has several limitations that should be considered while interpreting the findings. First, the retrospective observational design limited control over the completeness and uniformity of medico-legal documentation and precluded causal inference between associated factors and delayed reporting behavior. Second, as the study was conducted at a single tertiary-care referral center, referral patterns and healthcare accessibility may have contributed to selection bias, potentially underrepresenting survivors from remote rural settings or individuals who did not access medico-legal services. Consequently, the findings may not fully reflect the actual burden of underreported sexual assault cases within the community.

Additionally, reliance on retrospective record-based data may have resulted in incomplete documentation of certain behavioral, social, and contextual variables. Certain variables demonstrated inconsistent or inadequate documentation and were therefore excluded from regression modeling, which may have affected the comprehensiveness of the analysis. Socioeconomic categorization was based on retrospectively available record-based indicators, and a formally validated composite socioeconomic scale could not be applied because educational status was inconsistently documented across records.

Furthermore, some contextual variables related to delayed reporting, including family-related barriers and sociocultural pressures, may conceptually overlap with reporting behavior and should therefore be interpreted cautiously. Despite these limitations, the relatively large sample size and inclusion of socio-demographic, medico-legal, forensic, and reporting-related variables provide regionally relevant insights into sexual assault reporting patterns and medico-legal service utilization in Rajasthan.

Future research should focus on prospective, multi-center studies incorporating detailed behavioral, psychological, and forensic parameters. Qualitative studies exploring survivor experiences and barriers to reporting would further enhance understanding. Additionally, evaluation of targeted interventions aimed at reducing reporting delays and improving medico-legal outcomes is warranted.

## Conclusions

This present study provides a comprehensive analysis of sexual assault cases undergoing medico-legal examination at a tertiary care center in Rajasthan, highlighting important socio-demographic, clinical, and contextual patterns. The findings demonstrate a clear predominance of adolescent and unmarried victims, with a substantial proportion belonging to lower socioeconomic groups. A significant observation is that the majority of assaults were perpetrated by individuals known to the victim, underscoring the interpersonal nature of sexual violence. Delayed reporting emerged as a critical concern, with more than half of the victims presenting after 72 hours. Factors such as rural residence, younger age, involvement of known perpetrators, and family pressure were identified as significant factors associated with delayed reporting. These findings emphasize the influence of sociocultural and contextual determinants on reporting behavior and access to medico-legal services. Additionally, the low prevalence of detectable injuries and forensic evidence highlights the limitations of relying solely on physical findings in medico-legal evaluation. The study contributes to existing literature by integrating socio-demographic, behavioral, clinical, and legal variables and by applying multivariate analysis to identify independent predictors of delayed reporting. This multidimensional approach enhances understanding of the complex dynamics of sexual assault in the regional context.

From a practical perspective, the findings underscore the need for improving awareness, reducing stigma, and strengthening timely access to medico-legal and support services, particularly in underserved populations. Strengthening trauma-informed care and community-based interventions is essential to improve reporting and outcomes. In conclusion, addressing sexual assault requires coordinated, multi-sectoral efforts focusing on early reporting, survivor-centered care, and preventive strategies. Future research should adopt prospective and multi-center designs to further explore behavioral and systemic determinants and inform evidence-based policy interventions.
